# Local Regulatory Variation in *Saccharomyces cerevisiae*

**DOI:** 10.1371/journal.pgen.0010025

**Published:** 2005-08-19

**Authors:** James Ronald, Rachel B Brem, Jacqueline Whittle, Leonid Kruglyak

**Affiliations:** 1 Department of Genome Sciences, University of Washington, Seattle, Washington, United States of America; 2 Fred Hutchinson Cancer Research Center, Seattle, Washington, United States of America; 3 Howard Hughes Medical Institute, Seattle, Washington, United States of America; 4 Lewis–Sigler Institute for Integrative Genomics and Department of Ecology and Evolutionary Biology, Princeton University, Princeton, New Jersey, United States of America; Jackson Laboratory, United States of America

## Abstract

Naturally occurring sequence variation that affects gene expression is an important source of phenotypic differences among individuals within a species. We and others have previously shown that such regulatory variation can occur both at the same locus as the gene whose expression it affects (local regulatory variation) and elsewhere in the genome at *trans-*acting factors. Here we present a detailed analysis of genome-wide local regulatory variation in *Saccharomyces cerevisiae*. We used genetic linkage analysis to show that nearly a quarter of all yeast genes contain local regulatory variation between two divergent strains. We measured allele-specific expression in a diploid hybrid of the two strains for 77 genes showing strong self-linkage and found that in 52%–78% of these genes, local regulatory variation acts directly in *cis*. We also experimentally confirmed one example in which local regulatory variation in the gene *AMN1* acts in *trans* through a feedback loop. Genome-wide sequence analysis revealed that genes subject to local regulatory variation show increased polymorphism in the promoter regions, and that some but not all of this increase is due to polymorphisms in predicted transcription factor binding sites. Increased polymorphism was also found in the 3′ untranslated regions of these genes. These findings point to the importance of *cis*-acting variation, but also suggest that there is a diverse set of mechanisms through which local variation can affect gene expression levels.

## Synopsis

Variation in DNA sequences in and around a gene can contribute to differences between individuals by affecting the gene's expression. The authors have used a variety of methods to characterize this local DNA sequence variation on a large scale in two strains of the budding yeast *Saccharomyces cerevisiae*. Their results suggest that the expression levels of a sizeable fraction of genes are affected by local sequence variation. Many local variants alter the expression of only one of two copies of a gene in diploid hybrid yeast, but other local variants can affect both copies equally. The authors also found that sequence variation in particular regions of DNA near genes, both upstream and downstream of coding sequences and especially in transcription factor binding sites, is most likely to affect gene expression. These results provide a detailed view of local sequence variation that affects the expression of nearby genes in *S. cerevisiae*.

## Introduction

Much effort has recently been devoted to understanding the genetic basis of natural variation in gene expression levels. Linkage mapping, in which gene expression levels are treated as quantitative traits in linkage analysis, has been used to characterize the heritability of these expression traits and to identify the loci that control them [1–7]. Analysis of allele-specific expression (ASE), in which the relative amount of each allele in a diploid is assayed, has been used to identify genes with variation in *cis*-acting regulatory elements and to distinguish between *cis* and *trans* control [8–12]. These two approaches, linkage mapping and ASE analysis, provide distinct and complementary axes of information: positional and mechanistic, respectively.

We previously performed linkage analyses on gene expression levels in haploid segregants from a cross between two *Saccharomyces cerevisiae* strains (BY4716 [BY], isogenic to S288C, and RM11-1a [RM], a wild vineyard strain) [1]. We identified two types of linkages: those in which the expression level of a gene is linked to its own locus in the genome (“self-linkages”), and those in which the expression level is linked to a distinct locus elsewhere in the genome. The latter linkage indicates that variation at a distant locus acts in *trans* to affect expression of a gene [13]. In contrast, although self-linkage implies that local variation in the vicinity of the gene affects the expression of that gene, the mechanism through which that variation acts may be either *cis* or *trans,* under the classical definitions of the terms. For example, polymorphisms in the promoter region that affect chromatin structure or transcription factor binding sites, or polymorphisms in the coding sequence or 3′ untranslated region that affect mRNA stability, would be expected to act in *cis,* altering the abundance of the transcript in an allele-specific manner in a diploid [11]. Alternatively, amino acid changes within the coding sequence that affect the activity of the gene product, or codon usage changes that affect the level of protein, may lead to a change in gene expression either directly through autoregulation of the gene by its protein product or indirectly through a pathway of intermediates. Such local variation affecting the protein product, although present in only one allele in a heterozygous diploid, would act in *trans* to alter the expression of both alleles.

Here we performed a hypothesis-driven linkage analysis to improve the sensitivity with which genes subject to local regulatory variation are identified. We then used ASE measurements to estimate the fraction of local variation that acts mechanistically in *cis*. The observed high proportion of *cis*-acting effects in the genes assayed for ASE prompted us to perform a global analysis of polymorphism in genes with local regulatory variation, with emphasis on non-coding regions, to identify the signature of functional sequence differences. We found that genes with local regulatory variation are concentrated in areas of the genome that are highly polymorphic between the parent strains in both the genic and intergenic regions. We also found that genes showing evidence of local regulatory variation have further enrichment of polymorphisms in their promoter regions, in their 3′ untranslated regions, and specifically in predicted transcription factor binding sites, underscoring that fine-scale sequence variation in these regions is likely to have functional consequences.

## Results

### Many Genes Show Local Regulatory Variation

We previously reported self-linkage of 578 genes in a genome-wide analysis of a smaller cross (consisting of 86 segregants) between these strains [13]. To more sensitively identify genes subject to local regulatory variation, we tested for linkage only at the single marker closest to the gene in question, using previously reported gene expression and genotype data for 112 segregants from the same cross [14]. This hypothesis-driven approach reduced the number of statistical tests performed for each expression trait from a whole-genome scan of approximately 3,000 markers to a single-marker test and therefore increased the power to detect self-linkages. A total of 1,428 transcript levels (25% of the 5,727 transcripts tested) showed significant linkage at a permutation-based false discovery rate less than 0.05 (corresponding to a nominal *p*-value of 0.012). Multipoint linkage analysis showed that the gene encoding the linking transcript fell within the 1 logarithm of odds (LOD) support interval of the linkage peak in 92% of cases, and within the 2 LOD interval in 97% of cases. Based on genome-wide linkage results, we estimate that approximately 6% of true self-linkages may be due to polymorphisms in distinct *trans*-acting regulatory genes located close to their targets by chance (see Materials and Methods). Thus, a larger fraction of all genes (20%–25%) than previously reported contains local regulatory variation. The genes showing self-linkage, their effect size estimates, and their LOD support intervals are shown in Table S1.

### ASE of Genes with Local Regulatory Variation

In order to directly test whether local regulatory variation acts in *cis,* we assayed 77 genes showing self-linkage for the presence of ASE in a diploid hybrid of the two parent strains, BY and RM. These genes were chosen on the basis of showing highly significant self-linkage (*p* < 10^−8^) and at least a 1.2-fold difference in expression between segregants bearing the BY and RM alleles, such that no false positives and only one chance *trans* linkage due to a nearby gene were expected (see Materials and Methods). Of the 77 assayed genes, 44 (57%) showed ASE at a nominal *p*-value of less than 0.05 (Table S2). In only two of the 44 cases, ASE favored the allele associated by linkage analysis with lower expression. For comparison, we tested ASE in a control set of 16 genes that were selected because they showed heritable variation of equivalent effect size, with transcript levels linked to other loci in the genome, but without evidence of significant self-linkage. In this set of 16, we observed only two results with a nominal *p*-value of less than 0.05, a rate slightly higher but not significantly different from that expected by chance (Table S2).

We next sought an estimate of the total fraction of assayed genes with ASE, correcting for the fact that some true cases of ASE may not have reached a nominal *p*-value less than 0.05 in our experiment. To obtain this estimate, we used the method of Storey and Tibshirani [15], which considers the complete distribution of *p*-values to estimate the rate of true alternative hypotheses in a large set of statistical tests. This procedure estimated a true rate of ASE of 78% in the 77 genes tested. Such a high proportion of ASE is consistent with the results of Doss et al. [16], who showed that 18 of 28 self-linkages in a cross between two mouse strains had allele-specific effects, and those of Wittkopp et al. [11], who found that ASE was common in an F1 hybrid of *Drosophila melanogaster* and *D. simulans* among 29 genes with interspecific expression differences.

Our results also suggest that *trans*-acting local variation is likely to be responsible for a minority of the self-linkages tested. Indeed, a number of genes with self-linkage showed nearly equal expression of the two alleles in a diploid hybrid (Table S2). Although it has been argued that self-linkage without ASE is most likely due to a closely linked gene that happens to regulate the gene in question in *trans* [16], our linkage analyses suggest that such nearby regulators may not account for all local *trans*-acting effects in *S. cerevisiae* (see Materials and Methods). Instead, we believe that in some cases local *trans*-acting effects are best explained by a polymorphism in the gene itself that acts in *trans* through a feedback loop. For example, expression of the regulatory gene *AMN1* [17] showed strong self-linkage but weak ASE. Segregants that carry the BY allele of *AMN1* show a 2.2-fold increase in its expression relative to segregants that carry the RM allele, but in the diploid hybrid, the ratio of expression of the BY allele to expression of the RM allele is 1.12 ( *p* = 0.067; 95% confidence interval 0.99–1.27). We previously hypothesized that the functional polymorphism in *AMN1* is a single nucleotide substitution that leads to a missense amino acid change in the BY coding sequence at residue 368, replacing a highly conserved aspartic acid with valine [13]. The Amn1 protein has been proposed to indirectly negatively regulate itself as well as the daughter-specific transcriptional program, which includes the genes *DSE1* and *DSE2* [17]. *DSE1* and *DSE2* are upregulated 15.2- and 20.4-fold, respectively, in segregants bearing the BY allele at *AMN1,* consistent with the hypothesis that the negative regulator function of Amn1 is impaired in the BY strain. To determine whether the D368V amino acid change is the polymorphism that causes *AMN1* to show self-linkage, we engineered a BY strain carrying aspartic acid at residue 368 and measured gene expression levels using microarrays. We observed a 2.3-fold upregulation in the expression of *AMN1* in the original BY strain carrying the valine, relative to the engineered BY strain carrying aspartic acid at position 368. This result confirms that the coding mutation D368V is the predominant factor responsible for variation in expression of *AMN1*. In addition, we found that the original BY strain showed 9.7- and 15.3-fold upregulation of *DSE1* and *DSE2,* respectively, relative to the engineered strain carrying the aspartic acid; this further suggests that an aspartic acid at position 368 is sufficient to restore *trans*-regulatory function to Amn1 in the BY strain. This example directly illustrates that a change in protein sequence can lead to a difference in the encoding gene's expression, and that such a *trans*-acting mechanism affects both alleles equally through a feedback loop.

**Figure 1:**
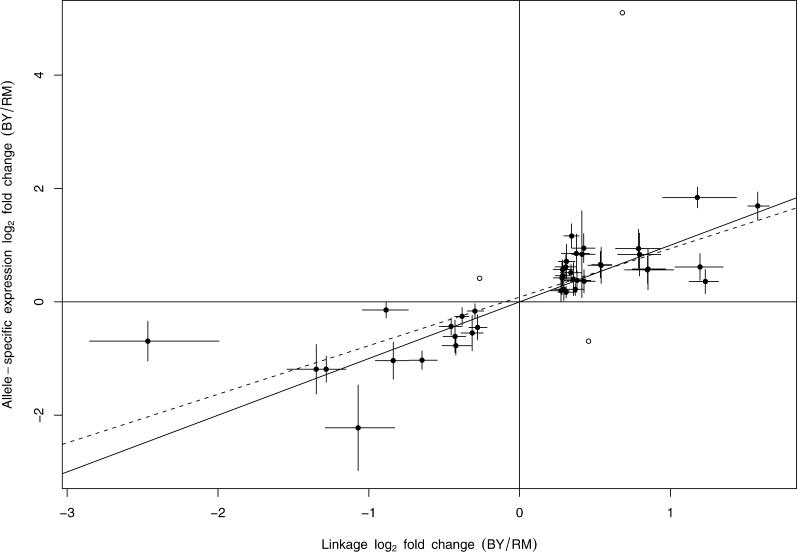
Comparison of Linkage and ASE Fold-Change Estimates Points represent the fold-change estimates from linkage analysis (horizontal axis) and from ASE experiments (vertical axis) for the 44 genes with nominally significant ASE (*p* < 0.05). Horizontal and vertical bars represent 95% confidence intervals. The solid line *(y* = *x)* represents equal fold-change estimates in the two experiments. The dashed line (*y* = 0.85*x* + 0.09, *R* ^2^ = 0.68) is the best fit, excluding one outlier and the two genes showing ASE favoring the opposite allele than that expected from linkage analysis (open circles).

Both *cis-*acting and *trans*-acting local polymorphisms could in principle affect expression of the same gene, so we next analyzed whether the expression changes in genes with *cis*-acting regulatory variation could be attributed primarily to this variation. Although the linkage study measured expression with microarrays and the ASE measurements were carried out by quantitative PCR, we noted that there was reasonable agreement between linkage results and ASE results in the fold-change estimates for the 44 nominally significant genes (Figure 1). Thus, for many of these genes, the linkage signal can be accounted for entirely by the polymorphisms producing ASE. Results for the 33 genes that were not significant for ASE, as well as the 16 controls, are shown in Figure S1.

Although we assayed a subset of yeast genes for ASE, our results may provide insight into the prevalence of ASE genome-wide. The distribution of self-linkage effect sizes of the 77 assayed genes was not different from that of all 446 genes with effect size greater than 1.2 (Kolmogorov–Smirnov test, *p* = 0.77), suggesting that the prevalence of ASE among the latter may be well represented by estimates from the assayed set. Indeed, the estimates may be appropriate for all genes with self-linkage irrespective of linkage effect size, as the prevalence of ASE among the genes assayed was not a function of this quantity: 21 of 39 genes with linkage effect size less than 1.34 and 23 of 38 genes with linkage effect size greater than 1.34 showed ASE at *p* < 0.05. In addition, the distribution of linkage effect sizes was indistinguishable among the 44 genes showing ASE at *p* < 0.05 and the remaining 33 genes (Kolmogorov–Smirnov test, *p* = 0.62). We therefore hypothesize that the subset of genes assayed here may be representative of self-linkages across the genome, and that a substantial fraction of all 1,428 self-linking genes is likely to show ASE due to the presence of *cis*-acting local regulatory variation.

### Genes with Local Regulatory Variation Map to Regions with Increased Polymorphism

**Figure 2:**
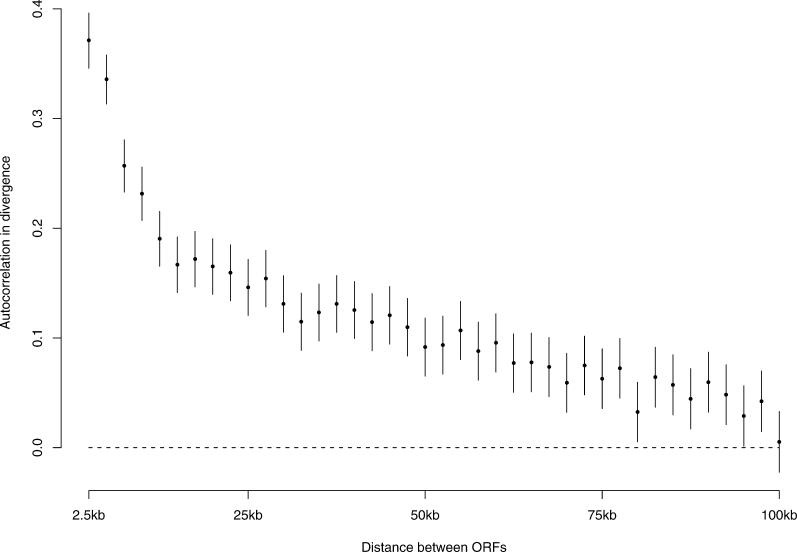
Autocorrelation in Divergence as a Function of Distance between Genes Each point indicates the correlation in the rate of substitution polymorphisms in the coding sequences of genes separated by at least *x* − 2.5 kb and at most *x* kb, for *x* = 2.5 kb, 5 kb, 7.5 kb, …, 100 kb. Correlations were similar for rates of substitution polymorphisms in intergenic regions (data not shown). ORFs, open reading frames.

**Figure 3:**
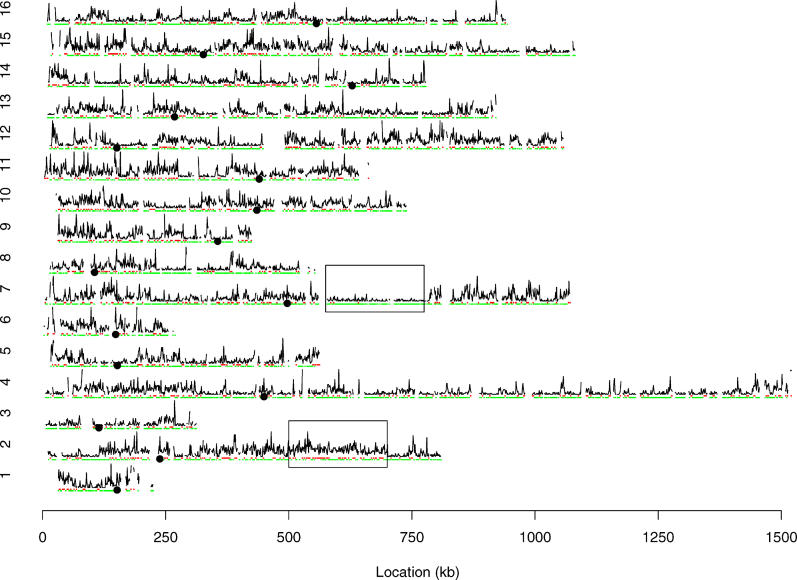
Rates of Substitution Polymorphisms between BY and RM Chromosome numbers are indicated on the left; large black circles represent centromere locations. Small points indicate gene locations: red, genes showing self-linkage; green, genes not showing self-linkage. The black jagged line represents the rate of substitution polymorphisms per 1,000 bp, with a maximum of 25 polymorphisms per 1,000 bases. The highly diverged Chromosome 2 region and relatively non-diverged Chromosome 7 region described in the text are indicated by boxes.

Because the ASE experiments in a selected set of genes led us to hypothesize that a substantial amount of local regulatory variation is due to *cis*-acting polymorphisms, we sought to analyze such variation genome-wide. We carried out a sequence comparison between the BY and RM strains for regions containing 5,182 genes with high-quality alignments between the genomes of the two strains. These 5,182 genes included 1,233 of the 1,428 genes showing self-linkage. Because the divergence in non-coding regions between BY and RM is approximately 0.005 (five polymorphisms per 1,000 bases) and most intergenic regions in *S. cerevisiae* are smaller than 1 kb, polymorphisms between these strains in non-coding regulatory regions are sufficiently infrequent that ascertainment of genes based on self-linkage should produce a detectable increase in divergence due to the presence of causative regulatory polymorphisms. We indeed observed a greater rate of polymorphisms in the upstream regions of the 1,233 genes showing self-linkage (0.0071, 95% confidence interval 0.0067–0.0075) compared to upstream regions of 3,949 genes without self-linkage (0.0040, 95% confidence interval 0.0038–0.0042). This increase was not limited to the region most likely to contain regulatory elements, but rather extended for at least 1 kb upstream (Figure S2). In fact, the polymorphism rate was also higher in the coding and downstream regions of genes showing self-linkage (0.0044 versus 0.0029 and 0.0069 versus 0.0040, respectively). These effects appear to be due in part to the correlation in the level of divergence over both short and long distances (Figure 2), with highly polymorphic regions tending to show a high density of genes with local regulatory variation (Figure 3).

For example, gross inspection of Figure 3 reveals a large region on Chromosome 7 that shows a low rate of polymorphism (approximately 0.6 polymorphisms per 1,000 bases over 200 kb) and, as a result, a low rate of genes showing self-linkage (three genes with self-linkage and 88 without). In contrast, an extended region on Chromosome 2 shows an elevated rate of polymorphism (approximately eight polymorphisms per 1,000 bases over 200 kb) and a large number of genes showing self-linkage (46 genes with self-linkage and 52 without). We found that the Chromosome 2 region showed a much higher polymorphism rate than the Chromosome 7 region in a comparison of BY and YJM789 [18] (a wild strain approximately as divergent from BY as RM), and a comparison of YJM789 and RM yielded a similar result, but essentially no difference in divergence was found between *S. cerevisiae* and *S. paradoxus* (data not shown). Thus, the heterogeneous pattern of divergence in these two regions apparently occurred in the *S. cerevisiae* lineage, but is not unique to a single strain. This is consistent with previous work showing extended regions of higher or lower diversity between yeast strains [19], presumably as a result of the complex, stochastic interbreeding and recombination histories of yeast strains that have led to differences in time to the most recent common ancestor for different chromosomal regions. The effects of such forces appear to be absent when more distantly related yeast genomes separated by a species barrier, such as *S. cerevisiae* and *S. paradoxus,* are compared [20].

**Figure 4:**
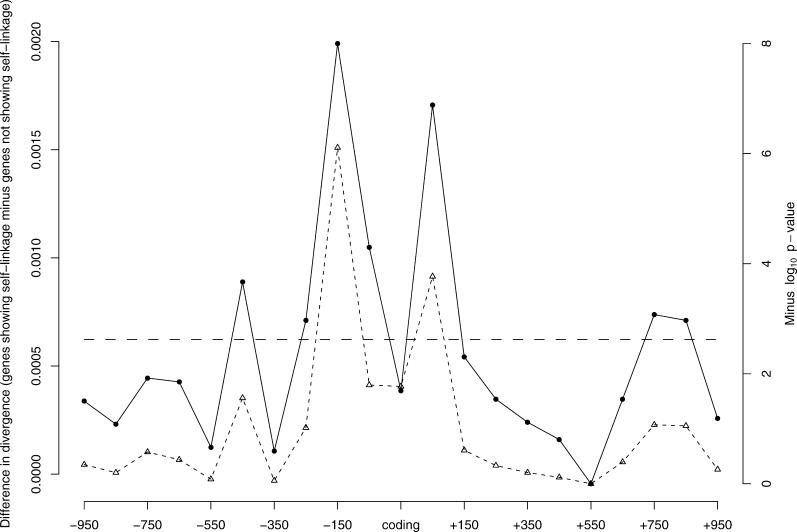
Increased Divergence in the Promoter Region and 3′ Untranslated Region in Genes Showing Self-Linkage The numbers of substitution SNPs are counted over each 100-bp bin centered at the position from translation start (or from translation stop for downstream bins) indicated on the *x*-axis, with the exception of the coding sequence, which is treated as a single bin. Solid line: the difference in inter-strain divergence between 1,125 position-matched pairs of genes, calculated by subtracting the average divergence in genes without self-linkage from the average divergence in genes with self-linkage (the left *y-*axis indicates difference in divergence in substitutions per basepair). Dotted line: −log_10_ *p*-values from logistic regression of self-linkage status on SNP rate in each bin independently, for the 1,125 pairs of genes (the right y-axis indicates −log_10_[*p*]); the dashed line shows *p* = 0.0024 (*p* = 0.05 after a Bonferroni correction for the 21 bins tested).

A higher rate of local regulatory variation (and hence a higher rate of genes showing self-linkage) is expected in more polymorphic regions of the genome. Such a result was also suggested by Hubner et al. [7] and observed by Doss et al. [16]. However, we were interested in whether we could use an enrichment for polymorphisms in specific regions of the genes (for example, likely regulatory regions) to identify those regions most likely to carry causative regulatory variants. Therefore, we sought to correct for the observed correlation in divergence and the resulting elevated rate of polymorphisms across entire genes, including upstream and downstream intergenic regions (Figure S2). We employed two approaches to do so. In both, we counted the number of substitution single nucleotide polymorphisms (SNPs) in each 100-bp bin from 1,000 bp upstream to translation start, treating each coding sequence as a single bin, and in each 100-bp bin from translation stop to 1,000 bp downstream. In the first approach, we performed logistic regression using all bins simultaneously in the model, estimating the significance of each bin conditional on all others; any overall, nonindependent elevation of polymorphism is factored out by this procedure (Figure S2). In the second approach, we directly matched genes by location, analyzing only those 1,125 genes with self-linkage that could be paired with exactly one unique gene without self-linkage located as close as possible but no more that 5,000 bp away. Both approaches showed significantly increased polymorphism, both upstream of translation start and downstream of translation stop, in genes showing self-linkage (Figure 4). The strongest enrichment for polymorphisms was found in the upstream region from 101 to 200 bp upstream of start (Figure 4). The enrichment in the downstream 3′ untranslated regions was not due to overlap with upstream regions of adjacent genes (see Materials and Methods; Figure S3). Thus, the increased polymorphism in genes with self-linkage in upstream and downstream regions is likely to reflect different classes of functional regulatory variation rather than local differences in the level of divergence.

### Enrichment of Polymorphisms in Transcription Factor Binding Sites

Our finding that ascertainment based on self-linkage most strongly enriches for polymorphisms from 101 to 200 bp upstream of translation start is consistent with the results of Cliften et al. [21], who found that intergenic conservation across six *Saccharomyces* species is highest in this region, and with the results of Harbison et al. [22], who found that transcription factor binding site occupancy is highest in this region. We therefore sought to analyze whether polymorphisms in this critical upstream region in genes showing self-linkage were more likely to be located in predicted transcription factor binding sites based on the dataset of Harbison et al. [22]. In order to control for both the increased rate of polymorphism in the upstream regions of genes with self-linkage and the decreased rate of polymorphism in predicted transcription factor binding sites due to negative selection on these functional sites, we performed chi-squared tests comparing the number of substitution SNPs in transcription factor binding sites versus non-sites in the upstream regions of genes with self-linkage versus genes without self-linkage.

This analysis showed that predicted transcription factor binding sites in the upstream regions of genes with self-linkage were modestly enriched for polymorphisms relative to surrounding bases (Table 1). When all genes showing self-linkage were analyzed, there was a trend for the enrichment to be greater at predicted sites that were conserved between species, and greatest at predicted sites that are bound by transcription factors in vivo [22]. This trend grew stronger when we compared those genes that showed self-linkage with fold-changes greater than 1.2 (approximately the 75th percentile of fold-change estimates) against genes without self-linkage. In this comparison, sites conserved in at least two additional species showed a 1.87-fold enrichment in polymorphisms (*p* = 0.021), while those with the strongest evidence of transcription factor binding showed a 3.73-fold enrichment (*p* = 0.00041). These results suggest that polymorphisms in conserved or bound sites tend to lead to larger changes in transcript abundance, but that polymorphisms in nonconserved sites may also contribute to variation in gene expression levels. In support of this finding, a functional role for nonconserved transcription factor binding sites was also shown by Doniger et al [23].

**Table 1:**
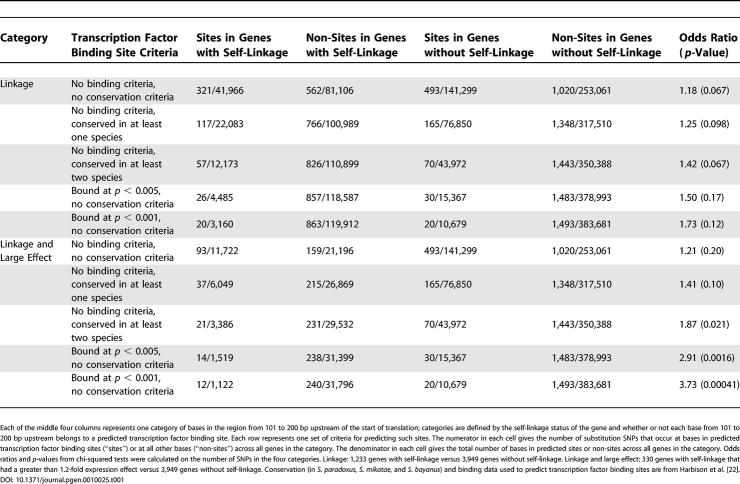
Polymorphisms in Transcription Factor Binding Sites

Each of the middle four columns represents one category of bases in the region from 101 to 200 bp upstream of the start of translation; categories are defined by the self-linkage status of the gene and whether or not each base from 101 to 200 bp upstream belongs to a predicted transcription factor binding site. Each row represents one set of criteria for predicting such sites. The numerator in each cell gives the number of substitution SNPs that occur at bases in predicted transcription factor binding sites (“sites”) or at all other bases (“non-sites”) across all genes in the category. The denominator in each cell gives the total number of bases in predicted sites or non-sites across all genes in the category. Odds ratios and *p*-values from chi-squared tests were calculated on the number of SNPs in the four categories. Linkage: 1,233 genes with self-linkage versus 3,949 genes without self-linkage. Linkage and large effect: 330 genes with self-linkage that had a greater than 1.2-fold expression effect versus 3,949 genes without self-linkage. Conservation (in *S. paradoxus, S. mikatae,* and *S. bayanus*) and binding data used to predict transcription factor binding sites are from Harbison et al. [22].

If some sites that do not bind transcription factors contain other functional regulatory sequences, these sites may also show increased polymorphism in genes with self-linkage. Thus, our comparison of transcription factor binding sites to the surrounding sequence may provide a conservative estimate of the importance of variation in transcription factor binding sites relative to truly neutral sequence. Therefore, we also compared the rate of polymorphisms in transcription factor binding sites to the rate of synonymous polymorphisms in the coding sequence. This analysis showed a more pronounced enrichment of polymorphisms in transcription factor binding sites in genes showing self-linkage, and also showed a tendency for increased polymorphism at sites 101–200 bp upstream that are not predicted to fall within transcription factor binding sites (Table S3).

## Discussion

We have found that nearly a quarter of yeast genes are affected by local regulatory variation between two strains. In our efforts to characterize fine-scale genetic variation that affects the expression level of nearby genes, we found that among a set of 77 genes showing strong self-linkage, most of these genetic changes act in *cis,* implying that they directly affect message abundance through changes in the rate of transcription or post-transcriptional regulation. A similar finding in the mouse [16] suggests that this observation is likely to be general. A high rate of *cis*-regulatory variation among all self-linking genes is supported by the observation of a highly significant increase in polymorphism in the promoter regions of genes with local regulatory variation, and a further increase in polymorphism in predicted transcription factor binding sites. The enrichment of polymorphisms in motifs discovered by cross-species conservation and binding site occupancy [22] suggests that variation in these sites is associated with changes in gene expression and, conversely, that discovery of regulatory polymorphisms may aid in the annotation of non-coding regulatory regions.

Although we found a global enrichment of polymorphisms in predicted transcription factor binding sites of genes with local regulatory variation, the effect was modest, and on a fine scale the pattern was complex. Even within the critical region 101–200 bp upstream thought to contain the highest density of transcription factor binding sites, genes with local regulatory variation showed an enrichment for polymorphisms at positions that are not predicted sites even by liberal criteria. Several explanations may account for this. First, we noticed that the rate of polymorphism between the two strains was non-uniform across the genome, with correlation in the level of divergence over both short and long distances. Thus, even in the analyses conditioned on local divergence, it is possible that some enrichment of polymorphism is due to an increased level of divergence rather than ascertainment based on functional significance. Second, it is likely that additional transcription factor binding sites in these genes exist, but that these sites have escaped detection because of their poor conservation across species, their low occupancy, or their noncanonical sequences. Finally, it is also possible that the increased level of polymorphism in the upstream region of genes with local regulatory variation may represent the signature of functionally important sites that participate in transcriptional control but are not directly involved in transcription factor binding. Indeed, Doniger et al. [23] argued that less than half of the functional constraint on *Saccharomyces* intergenic sequence could be attributed to predicted transcription factor binding sites based on known motifs, further suggesting that additional regulatory sequences remain to be discovered.

We also found increased polymorphism in the 3′ untranslated regions of genes with local regulatory variation that could not be explained by overlap with the promoter regions of adjacent genes, suggesting that sequence variants in this region can alter expression. Complex but non-random patterns of sequence conservation and composition have been observed in the 3′ untranslated regions of yeast genes, with significantly lower conservation in the 30 bases immediately downstream of translation stop and increased conservation in the subsequent 70 bases [24]. In addition, precision in the genome-wide control of mRNA half-life [25], which may involve the binding of Puf proteins to the 3′ UTR of mRNAs [26], further suggests that sequence signals besides those involved in transcriptional initiation play active roles in regulating transcript abundance.

A sizable minority of the genes with local regulatory variation that were assayed for ASE failed to show evidence of *cis*-acting variants. We also noted that genes showing self-linkage tended to have more polymorphisms in their coding sequences than genes not showing self-linkage. Although this increased polymorphism was not elevated above the baseline increase in divergence in genes showing self-linkage, more focused analyses of coding sequence polymorphisms may reveal changes in the gene product, which acts in *trans* to influence expression through autoregulation or feedback control, possibly indirectly through a pathway of mediators. One concrete example of such *trans*-acting local variation is the D368V amino acid substitution in Amn1. Another possible source of *trans*-acting variation in genes showing strong self-linkage is polymorphism in a different nearby gene that regulates the gene in question [16]. Our analyses suggest that this source can account for only a minority of self-linkages in our data.

Our results suggest that polymorphisms in the vicinity of a gene can affect its transcription level through a variety of mechanisms, with alteration of transcription factor binding sites being only one. Although this underscores challenges both in determining functional polymorphisms and in characterizing gene regulation in *S. cerevisiae,* unbiased identification of local regulatory variation through linkage analysis of expression levels will help to refine and validate currently proposed sets of regulatory motifs and will prompt exploration of novel classes of regulatory elements.

## Materials and Methods

### Linkage analysis and effect size estimates.

Linkage analysis and permutation tests were done as described [1] with genotypes and phenotypes from Brem and Kruglyak [14], except that only a single marker per transcript, the one closest to the gene's start site, was tested. A *p*-value of 0.012 corresponded to a false discovery rate less than 0.05 for the 5,727 transcripts tested. Thus, of the 1,428 transcripts showing significant self-linkage, 1,357 were expected to be true positives. The effect size of each self-linkage was computed as 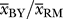 , where 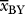 represents the mean expression level of the self-linking gene across all segregants bearing the BY allele at the marker closest to the gene, and 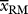 , represents the analogous mean taken with the RM allele. We obtained confidence intervals for the effect sizes by bootstrap resampling from the 112 segregants and taking the middle 95 of 100 fold-change estimates based on these resampled datasets.

The expression level of a transcript may show significant linkage to the location of its encoding gene for at least two reasons. The linking transcript level may vary because of a mutation in the coding sequence or regulatory region of its encoding gene. Alternatively, the linking transcript level may vary because of polymorphism in a neighboring regulatory gene acting in *trans*. We addressed the distinction between these scenarios in two ways. First, we performed nonparametric multipoint linkage analysis at 5-cM intervals using R/qtl [27] to define the LOD support interval for the highest peak on the respective chromosomes of each of the 1,428 transcripts showing self-linkage. For 1,313 and 1,380 of these transcripts, the encoding genes fell within the 1 LOD and 2 LOD support intervals, respectively. The observed data are in reasonable agreement with the theoretical expectations that the causative underlying polymorphism should be contained within the 1 LOD and 2 LOD support intervals approximately 90% and 99% of the time, respectively [28]. Next, we sought a direct estimate of the proportion of transcripts whose self-linkage was caused by a neighboring regulator. This proportion is related to the frequency of *trans*-acting regulators of transcript levels in the yeast genome. Therefore, we estimated the number of linkages across all transcripts that are due to *trans*-acting regulators. We selected a single marker for each transcript at random but not within 100 kb of the start site of the gene in question, and determined the number of such marker–transcript pairs that showed linkage at *p* < 0.012. We observed 160 significant linkages across all transcripts, 73 of which are expected to be false positives based on permutation tests. This suggests that testing a randomly chosen marker for each transcript across all transcripts is expected to yield 87 true positive linkages due to the presence of polymorphic *trans*-acting regulators near the markers. If such *trans*-acting regulators are distributed uniformly throughout the genome with respect to their targets, it follows that 87 of the 1,357 expected true positive self-linking transcripts in our data are due to polymorphisms in *trans*-acting regulators near the genes encoding the transcripts. In contrast, the remaining 94% of true positive self-linkages (1,270 of 1,357) are due to causative polymorphisms in the genes encoding the transcripts. Thus, 1,270 of 5,727 genes (22%) are expected to represent true self-linkages due to polymorphisms in the encoding genes. For the 77 genes with effect sizes greater than 1.2 chosen for direct tests of *cis*-acting variation by ASE, the significance level was more stringent (*p* < 10^−8^), such that no false positives were expected among the set of significant linkages. With these criteria (fold-change > 1.2 and *p* < 10^−8^), 224 self-linkages were identified with the single marker closest to the start site, while only three linkages were identified when the single marker was chosen at random as above. Thus, we expect 1.3% of self-linkages identified at this significance level (three out of 224), or one of the 77 tested, to be due to polymorphisms in nearby *trans*-acting regulators distinct from the gene in question.

The above analysis assumes that *trans*-acting regulators are distributed uniformly throughout the genome with respect to their targets. In the yeast genome, there is little evidence for strong deviation from this model. Approximately 19% of adjacent gene pairs show common function as opposed to 14% of random pairs [29], and less than 10% of adjacent pairs show correlated gene expression [30]. Thus, our estimate for the prevalence of *trans*-acting regulators falling near genes with self-linking transcript levels is likely to be reasonably accurate, and such neighboring factors are unlikely to be responsible for the majority of self-linkages we studied here.

### ASE measurements.

We used the TaqMan (Applied Biosystems, Foster City, California, United States) genotyping system in real-time quantitative PCR experiments to assay for the presence of ASE in diploid hybrids of BY and RM. Primer and probe sequences for the TaqMan assays are available on request. Genomic DNA and mRNA were extracted from four independent diploid cultures. In addition, we made two technical replicates each of 2:1, 1.5:1, 1.2:1, 1:1.2, 1:1.5, and 1:2 mixtures of each allele from the same extraction of BY and RM genomic DNA. This standard curve allowed us to identify outliers and assess the performance of each assay and to estimate the fold-change. It also suggested that we could reliably detect a 1.2-fold difference in the expression of each allele. For each sample, the logarithm of the fold-change in the amount of each allele present was estimated by the difference in cycle number at which the FAM and VIC dye intensities crossed the threshold intensity. We tested for the presence of ASE using a *t*-test to compare the diploid cDNA to the diploid genomic DNA and used the *t* statistic to form confidence intervals for the fold-change. In order to avoid underestimating the extent of ASE in these 77 genes because of the possibility of low statistical power in our four-sample versus four-sample *t*-test, we estimated 1 − π_0_, the rate of true alternative hypotheses, by the method of Storey and Tibshirani [15]. The estimate of 1 − π_0_ converged to a stable value (0.78) for maximum values of the tuning parameter ranging from 0.4 to 0.75. We observed 44 significant tests out of the expected 60 truly alternative tests estimated by 1 − π_0_, suggesting that our experiments had reasonable power (approximately 70%) to detect ASE at a *p*-value of less than 0.05.

### Effects of Amn1 polymorphism.

Linkage results for *DSE1*/YER124C and *DSE2*/YHR143W were from Brem and Kruglyak [14], and effect sizes of *AMN1* polymorphism were calculated as above. To test the effect of variation at amino acid 368, the D368 variant of *AMN1* was engineered into the S288c derivative JW2 (*MATα*, ura3Δ0, clonNAT+ downstream of *GPA1*) by the two-step gene replacement method [31]. Briefly, an integrating *URA3* plasmid carrying the D368 allele was introduced at the endogenous *AMN1* locus, resulting in a duplication of *AMN1*. Selection with 5FOA resulted in colonies that had lost the *URA3* marker and one of the copies of *AMN1*. The allele was determined by sequencing. Expression arrays were performed as by Yvert et al. [13], except that the reference sample was a 1:1 mixture of RNA from the BY and RM strains. Transcriptional effects given in the text represent the ratio between the wild-type control and allele-replaced strains.

### Sequence analysis.

The BY sequence was obtained from the *Saccharomyces* Genome Database (http://www.yeastgenome.org). The RM and YJM789 (version 2) whole-genome assemblies were obtained from the Broad Institute (http://www.broad.mit.edu/annotation/fungi/fgi/) and the Stanford Genome Technology Center (http://med.stanford.edu/sgtc/research/yjm789.html), respectively. We used CROSSMATCH (http://bozeman.mbt.washington.edu/phrap.docs/phrap.html) to identify a stringent set of ORFs in RM and in BY that were reciprocal best matches with at least 98% identity. We then aligned these sequences using CLUSTALW [32] and annotated the coding sequence and predicted transcription factor binding sites using data from the *Saccharomyces* Genome Database and supplementary data from Harbison et al. [22] (http://jura.wi.mit.edu/fraenkel/download/). Of the 5,727 ORFs in the linkage analyses, 5,182 met the criteria for high-confidence sequence alignment, 1,233 of which showed self-linkage.

### Statistical analyses.

All statistical analyses were performed using R [33]. Logistic regression analyses were performed using the number of substitution SNPs in each 100-bp bin as independent variables and the self-linkage status of each gene (zero if the gene did not show self-linkage, one if the gene showed self-linkage) as the dependent variable. The coding sequence was treated as a single bin. We set a ceiling of ten substitution SNPs per 100-bp bin to control for outliers due to low-quality subregions of alignments. For each bin, this ceiling affected no more than five of 2,250 genes total. The *p*-value for each bin was obtained separately by performing logistic regression to estimate the predictive value of a model containing that bin only. The *p*-value for each bin in the conditional logistic regression was obtained by estimating the additional predictive value of a model including all 21 bins relative to a null model containing the remaining 20 bins.

### Corrections for overlapping intergenic regions.

Because intergenic regulatory regions overlap between neighboring genes in the compact *S. cerevisiae* genome, we tested whether the apparently increased rate of polymorphism in the 3′ untranslated region could be explained by polymorphisms located in the promoter region of neighboring genes with self-linkage. We used two related approaches, one based on simulation and the other based on an expectation maximization (EM) algorithm, to estimate the rate of polymorphisms attributable to each bin and the rate attributable to overlapping bins of adjacent genes, conditional on the self-linkage status of the gene and its neighbors.

For the first correction method, we simulated the expected number of SNPs in each bin according to the specified underlying divergence for that bin and according to the divergence of overlapping bins as follows:


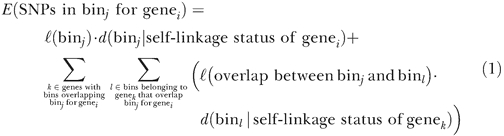


where 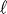 is the length of the quantity in parentheses and *d* is the divergence (per-base probability of substitution polymorphism) for that bin, given that it belongs to a gene showing self-linkage or a gene not showing self-linkage. We then minimized the difference between the expected number of SNPs per bin and the observed numbers of SNPs in each bin in the actual data over the parameters *d_ij_,* where


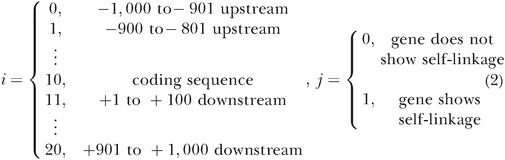


We chose initial values for *d_ij_* such that all bins had the same divergence both in genes showing self-linkage and genes not showing self-linkage, but the values of *d_ij_* that minimized the difference between the expected and observed data were similar over several different sets of initial values.

For the second correction method, we used an approach based on EM. For each SNP in the observed data, we assigned a fractional count to each bin into which the SNP fell as follows:


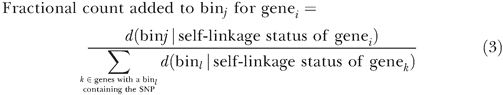


where *d* is the divergence for the bin. Initially, the *d_ij_* values were taken to be the observed divergence in each bin for genes showing self-linkage and genes not showing self-linkage. After each iteration, the *d_ij_* values were updated by calculating new values based on the fractional counts for each SNP. The choice of starting values had negligible impact on the final *d_ij_* obtained at convergence. Note that this approach differs somewhat from the simulation-based method in that, rather than specifying an underlying pattern of divergence and determining what distribution of SNPs it would produce, the EM approach takes the observed distribution of SNPs and estimates what underlying pattern of divergence would be most likely to produce it.

Both of the above procedures suggested that the spacing between adjacent genes is variable enough that no specific, artifactual enrichment of polymorphisms is produced in any single bin (Figure S3). As a final test, we repeated the logistic regression analysis across four separate subsets of the 2,250 genes: only those 1,057 genes whose start was more than 1,000 bases from the nearest start of any other gene, 1,065 genes whose start was more than 1,000 bases from the nearest stop, 925 genes whose stop was more than 1,000 bases from the nearest start, and 981 genes whose stop was more than 1,000 bases from the nearest stop of another gene. In spite of the reductions in power incurred by discarding much of the data for these analyses, the region from 101 to 200 bp upstream of translation start and the region from 1 to 100 bp downstream of translation stop continued to show significantly increased divergence in all analyses (Figure S3).

## Supporting information

Figure S1 Comparison of Linkage and ASE Fold-Change Estimates Points represent the fold-change estimates from linkage analysis (horizontal axis) and from ASE experiments (vertical axis). Horizontal and vertical lines on each point give the 95% confidence intervals. The solid diagonal line (*y* = *x*) represents equal fold-change estimates in the two experiments.
(A) Strong self-linkage is shown in 33 genes with ASE *p* > 0.05. The dashed line gives the line of best fit (*y* = 0.48*x* − 0.036; *p*-value for slope = 10^−8^). Note that the slope and the estimated π_0_ from the method of Storey and Tibshirani [15] suggest that a sizeable fraction of these 33 genes show ASE.
(B) Self-linkage *p* > 0.012 in 16 genes. The confidence intervals for several of the genes tested failed to overlap zero, suggesting that these genes may show weak self-linkage that did not meet our experiment-wide criterion of *p* < 0.012. The dashed line indicates the best fit (*y* = 0.33*x* − 0.008, *p*-value for slope = 0.37).
(16 KB EPS)

Figure S2 Increased Divergence across Extended Regions in Genes Showing Self-Linkage The *y*-axis represents −log_10_(*p*) from logistic regression of self-linkage status on SNP rate, for each 100-bp bin at the distance from translation start (or from translation stop for downstream bins) indicated on the *x*-axis. The coding sequence is treated as a single bin. Open circles connected by dashed lines: analysis of each bin separately, across all genes. Filled circles connected by solid lines: analysis of each bin conditional on SNP rates in all other bins, across all genes. Open triangles connected by dotted lines: analysis of each bin separately between 1,125 position-matched pairs of genes with and without self-linkage (see Figure 4). The dashed horizontal line indicates *p* = 0.0024 (*p* = 0.05 after a Bonferroni correction for the 21 bins tested).
(8 KB EPS)

Figure S3 Enrichment in Genes with Self-Linkage for Non-Coding Polymorphism Is Not an Artifact of Overlap between Intergenic Regions The *y-*axis indicates the difference in inter-strain divergence (substitutions per basepair) between 1,125 position-matched pairs of genes with and without self-linkage. Each point represents the divergence averaged over a 100-bp bin centered at the distance from translation start (or from translation stop for downstream bins) indicated on the *x*-axis. The coding sequence is treated as a single bin. Solid circles: difference in divergence estimated directly from observed data (see Figure 4). Triangles: difference in divergence corrected for overlap by the simulation-based approach (see Materials and Methods). Plus symbols: difference in divergence corrected for overlap by the EM-based approach (see Materials and Methods). Shading indicates regions with significantly increased divergence (*p* < 0.05) in genes showing self-linkage, across genes spaced at least 1,000 bp from one another.
(6 KB EPS)

Table S1 Linkage Analysis Results (235 KB TXT)

Table S2 ASE Results (7 KB TXT)

Table S3 Comparison of Promoter Polymorphisms with Synonymous Polymorphisms The first column lists selection criteria for transcription factor binding sites predicted by Harbison et al. [22]. The next two columns list the numbers of substitution polymorphisms in transcription factor binding sites (numbers of substitution polymorphisms in all other bases not belonging to predicted sites, labeled as non-sites, are in parentheses) in the region from 101 to 200 bp upstream of translation start in genes showing self-linkage and genes not showing self-linkage, respectively. The next two columns show the numbers of substitution polymorphisms in synonymous sites in the coding sequences of genes showing self-linkage and genes not showing self-linkage, respectively. Boundaries of the coding sequences were determined by *Saccharomyces* Genome Database annotations. If a gap or premature stop codon was encountered in the RM sequence, all subsequent codons were ignored. Each row in the table represents one set of criteria for transcription factor binding site prediction (as in Table 1). In the last column, *p*-values are from a chi-squared test comparing the number of substitutions in “sites” and “non-sites” in genes with or without self-linkage to the number of synonymous substitutions in genes with or without self-linkage. Linkage: 1,233 genes with self-linkage versus 3,949 genes without self-linkage. Linkage and large effect: 330 genes with self-linkage that had a greater than 1.2-fold expression effect versus 3,949 genes without self-linkage.
(43 KB DOC)

## Abbreviations

ASE: allele-specific expression
BY: BY4716
EM: expectation maximization
LOD: logarithm of odds
RM: RM11-1a
SNP: single nucleotide polymorphism
