# Supplementary material for: Local Regulatory Variation in Saccharomyces cerevisiae
Source: PLoS Genet. 2005 Aug 19;1(2):e25. doi: 10.1371/journal.pgen.0010025 (PMC1189075; doi:10.1371/journal.pgen.0010025)
Supplement: Supplementary file 1 [file pgen.0010025.st003.doc]

|  | Transcription factor bind site criteria | Sites in genes with self-linkage  (Non-sites) | Sites in genes without self-linkage  (Non-sites) | Synonymous substitutions in genes with self-linkage | Synonymous substitutions in genes without self-linkage | Sites *p*-value (Non-sites *p*-value) |
| --- | --- | --- | --- | --- | --- | --- |
| Linkage | No binding criteria, no conservation criteria | 321  (562) | 493  (1020) | 4597 | 9179 | 0.0004  (0.091) |
|  | No binding criteria, conserved in at least 1 species | 117  (766) | 165  (1348) | 4597 | 9179 | 0.005  (0.010) |
|  | No binding criteria, conserved in at least 2 species | 57  (826) | 70  (1443) | 4597 | 9179 | 0.008  (0.005) |
|  | Bound at *p* < 0.005, no conservation criteria | 26  (857) | 30  (1483) | 4597 | 9179 | 0.054  (0.002) |
|  | Bound at *p* < 0.001, no conservation criteria | 20  (863) | 20  (1493) | 4597 | 9179 | 0.040  (0.002) |
| Linkage and large effect | No binding criteria, no conservation criteria | 93  (159) | 493  (1020) | 1301 | 9179 | 0.017  (0.31) |
|  | No binding criteria, conserved in at least 1 species | 37  (215) | 165  (1348) | 1301 | 9179 | 0.016  (0.15) |
|  | No binding criteria, conserved in at least 2 species | 21  (231) | 70  (1443) | 1301 | 9179 | 0.004  (0.12) |
|  | Bound at *p* < 0.005, no conservation criteria | 14  (238) | 30  (1483) | 1301 | 9179 | 0.0003  (0.11) |
|  | Bound at *p* < 0.001, no conservation criteria | 12  (240) | 20  (1493) | 1301 | 9179 | 0.0003  (0.10) |
